# Supplementary material for: MetaRibo-Seq measures translation in microbiomes
Source: Nat Commun. 2020 Jun 29;11:3268. doi: 10.1038/s41467-020-17081-z (PMC7324362; doi:10.1038/s41467-020-17081-z)
Supplement: Supplementary file 10 — Supplementary Data 7 [file 41467_2020_17081_MOESM10_ESM.zip › File2/Confidence_High_Taxonomy/155299_out.krona.html]

Javascript must be enabled to view this page.

members
magnitude
magnitudeUnassigned
count
unassigned
taxon
rank

155299\_out

77

1

SRS017521\_contig\_number\_22904

2
superkingdom
76

phylum
201174
74

1760
class
74

2037
order
74

74
2049
family

74
1654
genus

1

SRS015395\_contig\_number\_contig-100\_1655.66999
species
1739450

1
species
712118

SRS016319\_contig\_number\_24230

1
1227263
species

SRS149492\_contig\_number\_8428


SRS011086\_contig\_number\_contig-100\_6084.54291.54291SRS013651\_contig\_number\_13909SRS013879\_contig\_number\_14478SRS014684\_contig\_number\_28830SRS014689\_contig\_number\_contig-100\_8906.41305SRS015038\_contig\_number\_contig-100\_537.35559SRS015057\_contig\_number\_16230SRS015941\_contig\_number\_26129SRS016086\_contig\_number\_21520SRS016474\_contig\_number\_contig-100\_20.111674SRS018679\_contig\_number\_6423SRS018739\_contig\_number\_28599SRS018875\_contig\_number\_28054SRS018923\_contig\_number\_567SRS020571\_contig\_number\_36376SRS020628\_contig\_number\_41659SRS021528\_contig\_number\_11396SRS022077\_contig\_number\_30373SRS024318\_contig\_number\_39232SRS024375\_contig\_number\_26303SRS043461\_contig\_number\_9448SRS043494\_contig\_number\_4292SRS044486\_contig\_number\_contig-100\_683.100240SRS048317\_contig\_number\_11921SRS051791\_contig\_number\_21233SRS055495\_contig\_number\_contig-100\_10759.10759SRS063621\_contig\_number\_3635SRS074970\_contig\_number\_19100SRS075813\_contig\_number\_12665SRS075870\_contig\_number\_contig-100\_803.168883SRS076951\_contig\_number\_contig-100\_4178.202973SRS076951\_contig\_number\_contig-100\_2680.229975SRS077016\_contig\_number\_4269SRS077239\_contig\_number\_23433SRS077300\_contig\_number\_contig-100\_2719.60934SRS077308\_contig\_number\_16970SRS077441\_contig\_number\_contig-100\_17.57810SRS077568\_contig\_number\_7407SRS077997\_contig\_number\_contig-100\_10893.64734SRS078677\_contig\_number\_9525SRS098498\_contig\_number\_38317SRS098704\_contig\_number\_6197SRS1041119\_contig\_number\_8225SRS104291\_contig\_number\_contig-100\_27177.27177SRS1055059\_contig\_number\_contig-100\_6780.80710SRS1055060\_contig\_number\_contig-100\_6597.269061SRS1055078\_contig\_number\_contig-100\_3635.3636SRS143354\_contig\_number\_5956SRS143604\_contig\_number\_10141SRS143678\_contig\_number\_contig-100\_3.103213SRS143879\_contig\_number\_27068SRS144014\_contig\_number\_19664SRS144090\_contig\_number\_16458SRS144194\_contig\_number\_13915SRS144644\_contig\_number\_24430SRS144644\_contig\_number\_contig-100\_5829.134577SRS146738\_contig\_number\_11887SRS146996\_contig\_number\_12586SRS147045\_contig\_number\_10178SRS147094\_contig\_number\_34671SRS147933\_contig\_number\_705SRS148150\_contig\_number\_16700SRS148303\_contig\_number\_contig-100\_9.85435SRS148361\_contig\_number\_6535SRS148408\_contig\_number\_12470SRS148724\_contig\_number\_11621SRS148724\_contig\_number\_contig-100\_2595.2596SRS149184\_contig\_number\_contig-100\_6721.108629SRS893274\_contig\_number\_contig-100\_2079.90752SRS893277\_contig\_number\_6155SRS893350\_contig\_number\_contig-100\_1374.163191
species
1739406
71

phylum
976
2

2
class
200643

2
order
171549

171552
family
2

2
577309
genus

2

SRS049712\_contig\_number\_contig-100\_1617.119059SRS105082\_contig\_number\_contig-100\_1326.112212
454154
species
